# Coping with extremes: the rumen transcriptome and microbiome co-regulate plateau adaptability of Xizang goat

**DOI:** 10.1186/s12864-024-10175-8

**Published:** 2024-03-07

**Authors:** Cheng Pan, Haiyan Li, Shehr Bano Mustafa, Cuomu Renqing, Zhenzhen Zhang, Jingjing Li, Tianzeng Song, Gaofu Wang, Wangsheng Zhao

**Affiliations:** 1 School of Life Science and Engineering, Southwest University of Science and Technology, 621000 Mianyang, Sichuan China; 2 Institute of Animal Science, Xizang Academy of Agricultural and Animal Husbandry Science, 850009 Lhasa, Xizang China; 3 Key Laboratory of Animal Genetics and Breeding on Xizang Plateau, Ministry of Agriculture and Rural Affairs, 850009 Lhasa, Xizang China; 4 Chongqing Academy of Animal Sciences, 402460 Chongqing, Rongchang China

**Keywords:** Xizang goat, Plateau adaptability, Rumen microbiota, Transcriptome, Climate

## Abstract

The interactions between the rumen microbiota and the host are crucial for the digestive and absorptive processes of ruminants, and they are heavily influenced by the climatic conditions of their habitat. Owing to the harsh conditions of the high-altitude habitat, little is known about how ruminants regulate the host transcriptome and the composition of their rumen microbiota. Using the model species of goats, we examined the variations in the rumen microbiota, transcriptome regulation, and climate of the environment between high altitude (Lhasa, Xizang; 3650 m) and low altitude (Chengdu, Sichuan, China; 500 m) goats. The results of 16 S rRNA sequencing revealed variations in the abundance, diversity, and composition of rumen microbiota. *Papillibacter*, *Quinella*, and *Saccharofermentans* were chosen as potential microbes for the adaptation of Xizang goats to the harsh climate of the plateau by the Spearman correlation study of climate and microbiota. Based on rumen transcriptome sequencing analysis, 244 genes were found to be differentially expressed between Xizang goats and low-altitude goats, with 127 genes showing up-regulation and 117 genes showing down-regulation. *SLC26A9*, *GPX3*, *ARRDC4*, and *COX1* were identified as potential candidates for plateau adaptation in Xizang goats. Moreover, the metabolism of fatty acids, arachidonic acids, pathway involving cytokines and their receptors could be essential for adaptation to plateau hypoxia and cold endurance. The expression of *GPX3*, a gene linked to plateau acclimatization in Xizang goats, was linked to the abundance of *Anaerovibrio*, and the expression of *SLC26A9* was linked to the quantity of *Selenomonas*, according to ruminal microbiota and host Spearman correlation analysis. Our findings imply that in order to adapt harsh plateau conditions, Xizang goats have evolved to maximize digestion and absorption as well as to have a rumen microbiota suitable for the composition of their diet.

**Supplementary Information:**

The online version contains supplementary material available at 10.1186/s12864-024-10175-8.

## Introduction

One of the first animals that humans domesticated was the goat [1]. In contrast to goats found in low altitudes, Xizang goats are exposed to harsh conditions, including high altitude, low oxygen, and intense UV radiation. To cope with these conditions, Xizang goats have evolved unique morphologies, physiological and biochemical characteristics, and genetic adaptations to withstand plateau stress [2–4].

The rumen, reticulum, abomasum, and omasum are the four chambers of a ruminant’s stomach [5]. The rumen, the largest chamber in ruminants, offers anaerobic conditions for microbial fermentation. This process is crucial for the breakdown of proteins, carbohydrates, starch and fats through anaerobic fermentation, and it also supplies the host with nutrients in the form of microbial proteins and volatile fatty acids (VFAs) [6]. With bacteria comprising 70% of all microorganisms, the rumen is rich in a variety of microorganisms, including fungi, bacteria, protozoa, and archaea. The rumen’s microbiota structure is closely related to immunological defenses, nutrition control, host growth and development, and modifications to the environment [7, 8]. According to certain studies, the predominant microorganisms in the high and low altitude regions differed significantly, and the proportion of bacteria that break down fiber was significantly higher in the high altitude than in the low altitude, while the proportion of bacteria that break down starch did not differ significantly [9]. These findings suggest that yak rumen microorganisms are somewhat adaptable to different altitudes. Another study investigated the forage fermentation and rumen microbial diversity in yaks raised to three, four, and five thousand meters above sea level. It was discovered that, as forage fermentation efficiency increased, rumen microbial diversity declined with altitude. This suggests that yak rumen microorganisms have adapted to alpine environments in tandem with their hosts [10]. The fundamental idea of evolutionary biology is genetic adaptability, which is widely accepted. The expression of genes involved in energy metabolism, intimately linked to the metabolic processes of rumen bacteria, is influenced by adaptive evolution in ruminants at high altitudes [11]. Highland Xizang sheep use rumen fermentation and gene interactions with epithelial cells to control immune system function and food intake [12]. Nevertheless, a study found that both endogenous and exogenous factors, including host immunity, metabolism, and dietary structure, had an impact on microbial diversity. However, these factors only explained 10–20% of the variation in microbial diversity [13, 14]. To better understand plateau adaptation in ruminants, a highly sought-after and crucial tool involves combining transcriptomic, epigenomic, metabolomic, and microbial polyomics.

Several studies have demonstrated how crucial it is to fully sequence the gastrointestinal microbiome and macro genome to correlate the results with the host genome, transcriptome, and metabolic profiles to uncover the pertinent patterns and mechanisms of the physiological properties of the host [15]. Functional connections between rumen bacteria and the host were found during the transcriptome and microbiome analysis of the rumen epithelium development in lambs [16]. These findings imply that microbes and the host work together to support the development of the rumen’s epithelium. To investigate the dynamics of rumen’s function, microbial colonization, and functional relationships between them during the first eight weeks of life, transcriptome and macroeconomic data from the goat rumen were synthesized [17]. The mechanisms by which the Xizang sheep’s rumen transcriptome, microbiome, and metabolome work in concert to regulate adaption during the cold season were discovered using a multi-omics approach [18]. Some reports have revealed the microbial composition and gene function of the gastrointestinal tract of ruminants on the Xizang Plateau, as well as the interaction of rumen microbes and their metabolites with the host [10, 11, 17]. But there are currently few reports on the study of choosing low-altitude goats and high-altitude Xizang goats for comparative analysis to explore the plateau acclimatization of the Xizang goat.

The purpose of this study was to learn more about the interactions that occur between the host transcriptome and rumen microorganisms throughout the acclimatization process on the plateau in Xizang goats. Thus, in this study, key genes regulating plateau acclimatization, interactions between rumen microbiota and the host transcriptome, and the major microbiota associated with plateau acclimatization in Xizang goats were explored using 16 S rRNA sequencing and rumen transcriptome (mRNA sequencing). High-altitude Xizang goats were used as test subjects, while low-altitude goats were chosen as the control group.

## Materials and methods

### Experimental design, sample, and climate data collection

High-altitude Xizang goats from Lhasa, Xizang Autonomous Region were selected as the experimental study subjects and low-altitude goats from Chengdu, Sichuan Province were used as the control group. Six rams each with similar body weight, good health condition, and similar day old were randomly selected respectively. The rumen fluid was collected from the goats with a gastric tube rumen sampler, filtered through gauze, and transferred to a 2 mL frozen storage tubes. The collected rumen fluid was quickly frozen in a liquid nitrogen tank brought back to the laboratory and stored at– 80 ℃ for later 16 S rRNA analysis. After slaughter, 1 cm^2^ of rumen tissue was collected and rinsed quickly with PBS, and the epithelial tissue was isolated and quickly stored in liquid nitrogen for subsequent extraction of total RNA. Climatic data were obtained from the website https://www.weather-atlas.com/zh.

### RNA extraction, transcriptome analysis, reverse transcription-quantitative PCR validation

Total RNA from rumen epithelial tissues of high-altitude Xizang goats and low-altitude goats was extracted by the Trizol method. After successful extraction, the RNA was added to 50µL of DEPC treated water to dissolve the RNA. Subsequently, total RNA was identified and quantified using NanoDrop and Agilent 2100 Bioanalyzer (Thermo Fisher Scientific, MA, USA). The mRNA was purified using Oligo(dT) magnetic beads.The purified mRNA was lysed into small fragments with fragmentation buffer at an appropriate temperature. The first strand of cDNA was then generated by reverse transcription using random hexamer primers and then second strand of cDNA was then synthesized.The end repair was then performed by incubation with the addition of a tail mix and RNA index adapter. cDNA fragments obtained by PCR amplification were used to purify the product with Ampure XP beads and then solubilized in ethidium bromide (EB) solution. The product was verified for quality control on an Agilent Technologies 2100 Bioanalyzer. The double-stranded PCR product obtained in the previous step was heat denatured and circulated through the clipboard oligonucleotide sequence to obtain the final library. Single-stranded circular DNA (ssCir DNA) was formatted into the final library. The constructed libraries were machine-sequenced using Illumina NovaseQ 6000 (San Diego). To verify the reproducibility of the gene expression data, six differentially expressed genes (DEGs) were randomly selected for reverse transcription-quantitative PCR (RT-qPCR) method from individual RNA samples initially extracted by RNA-seq. For primer information for these DEGs, see Table 1.

**Table 1:** Primers used for quantitative real-time PCR

Genes	Sequence(5’→3’)	Product size/bp
*GAPDH*	F: CCTGCCAAGTATGATGAGAT	117
	R: AGTGTCGCTGTTGAAGTC	
*SLC6A4*	F:GTATATGGCTGAGATGAGGAA	92
	R:CTATGGCTTCTGCGTATGT	
*ME1*	F: GAGCAAGCCATACAGAAGA	174
	R: AATCGCAGCAACTCCAAT	
*SUOX*	F:CCTTCTCTGGTGGTAACTC	124
	R:TATCTGCGTGGTGACTCT	
*GPX3*	F:ATTCGGTCTGGTCATTCTG	200
	R: GAGGACAGGAGTTCTTCAG	
*CPEB4*	F:TCTCCTACACCGTCTTCTT	107
	R:GGCGTTATTCCTCCATTCA	
*TKT*	F:ACGGAGAAGGCAGTAGAA	166
	R:CAGCACCAATCACAGTCA	

### DNA extraction and 16 S rRNA sequencing

Using the hexadecyl trimethyl ammonium bromide (CTAB) method, total genome DNA was isolated from the rumen fluid of Xizang goats raised at high altitudes and goats raised at low altitudes. On a 1% agarose gel, the concentration and purity of DNA were observed. Using sterile water, DNA was diluted to 1ng/L based on the concentration. 15 L of Phusion® High-Fidelity PCR Master Mix (New England Biolabs), 2 mM of forwards and reverse primers, and roughly 10 ng of template DNA were used in the PCR reactions. The process of thermal cycling involved a one-minute initial denaturation at 98 °C, thirty cycles of denaturation for ten seconds at 98 °C, thirty seconds of annealing at 50 °C, and thirty seconds of elongation at 72 °C. Lastly, 72 °C for five minutes. Combine the PCR products with an equal volume of 1 x loading buffer that contains SYB green, then run the electrophoresis on a 2% agarose gel to detect the results. Equidensity ratios were used to combine the PCR products. Next, a Qiagen Gel Extraction Kit (Qiagen, Germany) was used to purify a mixture of PCR products. After creating sequencing libraries using the TruSeq® DNA PCR-Free Sample Preparation Kit (Illumina, USA) in accordance with the manufacturer’s instructions, index codes were inserted.The Qubit@ 2.0 Fluorometer (Thermo Scientific) and Agilent Bioanalyzer 2100 system were used to evaluate the quality of the library. Ultimately, the library underwent sequencing on an Illumina NovaSeq device, yielding paired-end reads of 250 bp of length.

### Data analysis

Climate data was statistically analyzed using independent samples t-tests in IBM SPSS Statistics 21 software; Both the relationship between rumen bacteria and DEG related to rumen metabolite plateau adaptation and the correlation between rumen bacteria and climate were found using Spearman’s correlation analysis. *P* < 0.05 were considered statistically significant, *P* < 0.01 were considered highly significant, and *P* < 0.001 were considered extremely significant.

## Results

### Climate change in the subsistence environment of the Xizang mountains

**Fig. 1:**
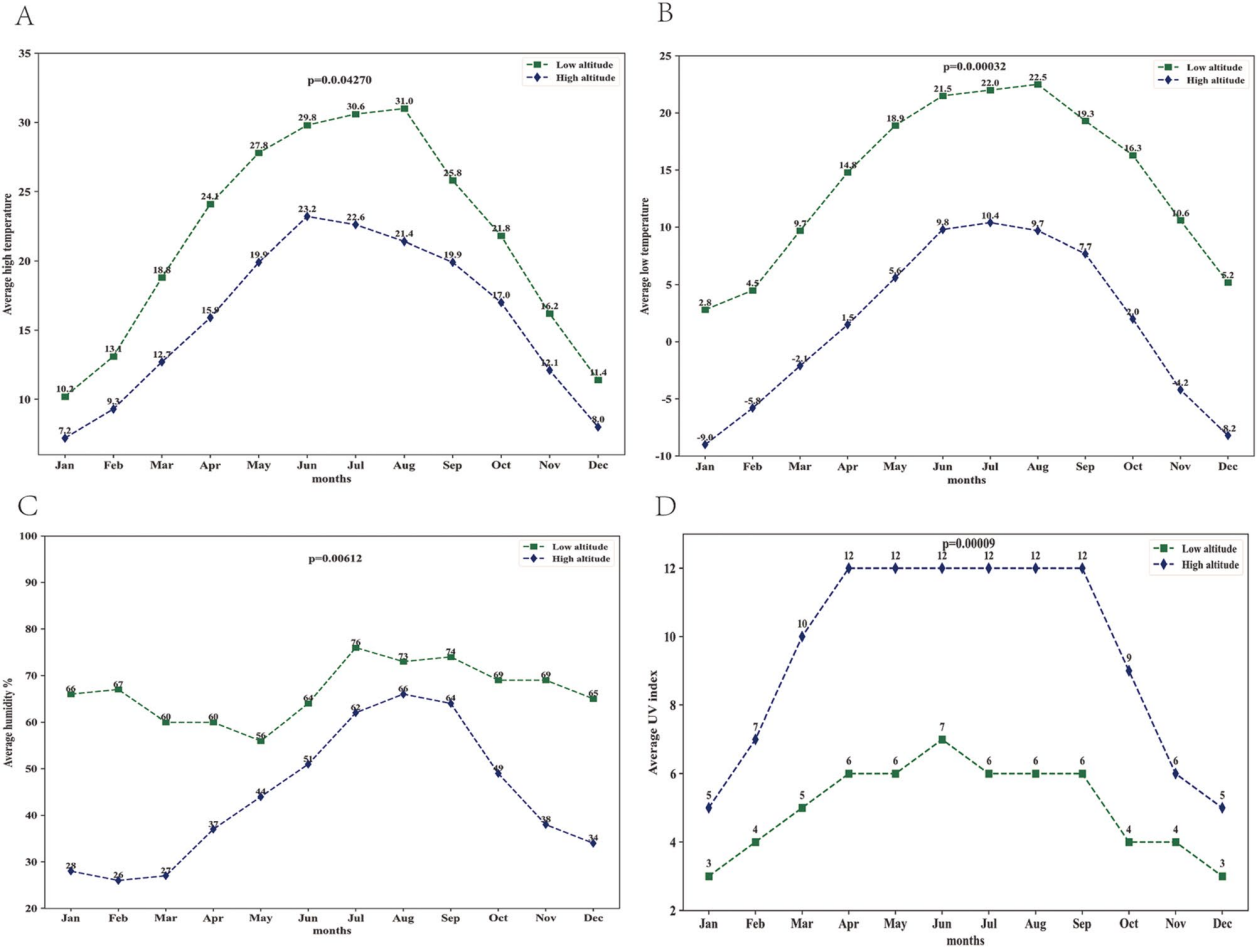
Comparison of climate change in the living environment of Xizang goats and low-altitude goats. (**A**) Comparison of changes in average high temperatures. (**B**) Comparison of changes in average low temperatures. (**C**) Comparison of changes in average humidity. (**D**) Comparison of changes in average UV index. Lhasa represents high altitude, and Chengdu represents low altitude

Xizang goats face harsher living conditions, including high temperatures, low oxygen levels, and intense UV radiation, compared to low-altitude goats. Figure 1(A)-(D) shows the major variation in climate between the high-altitude area (Lhasa) and low-altitude area (Chengdu). The average UV index was significantly lower in the low-altitude area. In the low-altitude region, average high and low temperatures as well as humidity were noticeably higher. This implies that Xizang goats might survive in dry, high-radiation, and extremely cold environments.

### Changes in *α* and *β* diversity of Rumen Microbiota in Xizang goats

**Fig. 2:**
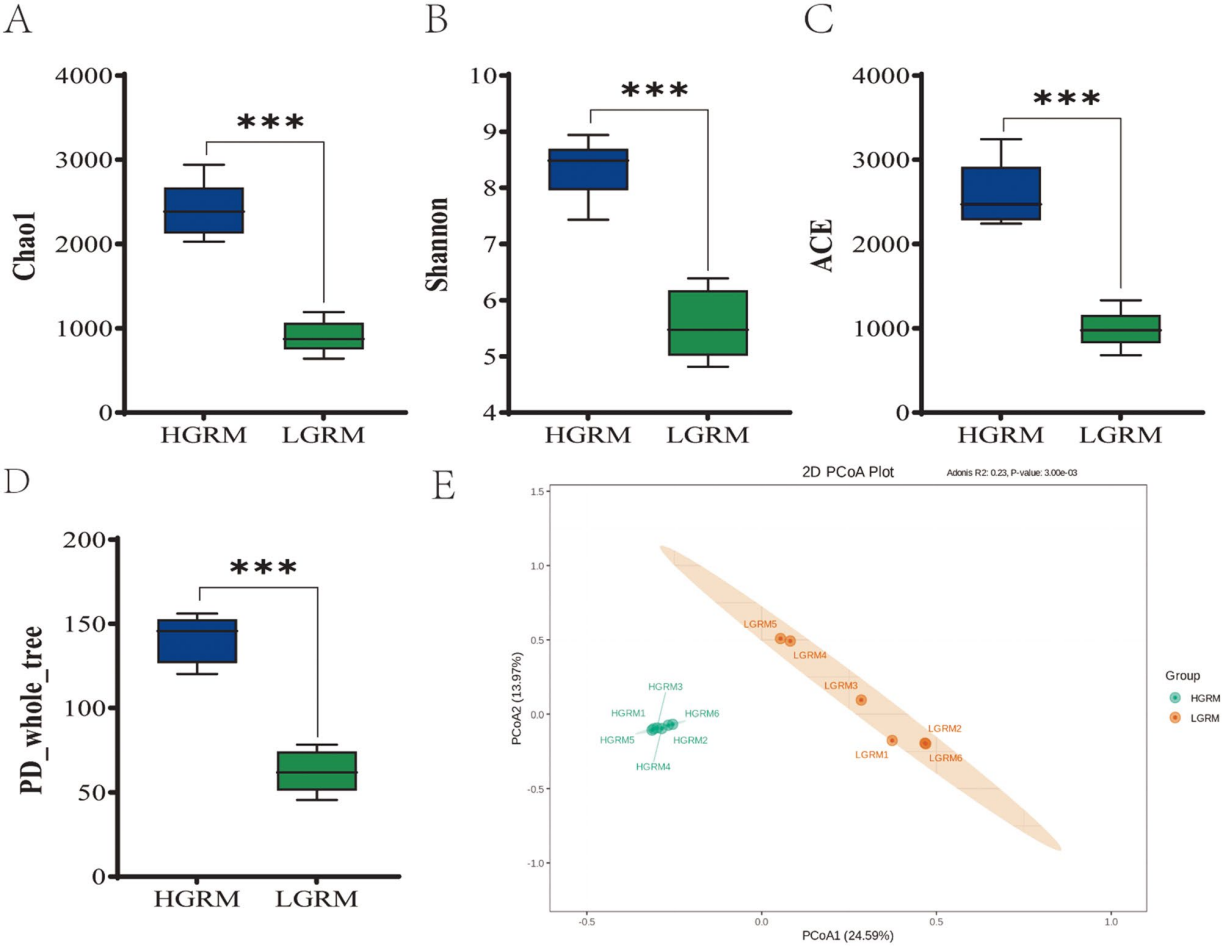
Comparison of changes in rumen microbiota *α* and *β* diversity in Xizang and low-altitude goats. (**A**) Chao1. (**B**) Shannon. (**C**) ACE. (**D**) PD-whole-tree. (**E**) PcoA. ⁎ denotes significant, ⁎⁎ denotes highly significant, ⁎⁎⁎ denotes extremely significant, Xizang goats represent HGRM, Low-altitude goats represent LGRM)

The Rarefaction Curve is a commonly used curve that describes the diversity of samples within a group. It directly reflects the appropriateness of the amount of sequencing data and indirectly reflects the diversity of species in the samples. In both the Xizang and low altitude goat groups, the Rarefaction Curve tended to flatten (Additional file 1), indicating that the quantity of sequencing data was asymptotically appropriate. The microbiota’s richness and diversity were primarily measured using the Chao1 index, Shannon index, PD_whole_tree, and ACE index. The *α* and *β* diversities of the goats’ rumen microbiota varied depending on their height. Figure 2(A-D) display the results of the study, which describes that the rumen microbiota of Xizang goats had considerably greater levels of the Chao1 index, Shannon index, PD_whole_tree, and ACE index (*P* < 0.01) than those of low-altitude goats. As shown in Fig. 2E, the PCoA analysis revealed a significant difference in *β* diversity between the low-altitude goats’ and Xizang goats’ rumen microbiota. The results indicated that the abundance and diversity of rumen microbiota of Xizang goats were significantly higher than those of low-altitude goats, suggesting that a higher abundance and diversity of microbiota may be favorable for survival in the host plateau environment.

### Changes in Rumen Microbiota composition in Xizang goats

**Fig. 3:**
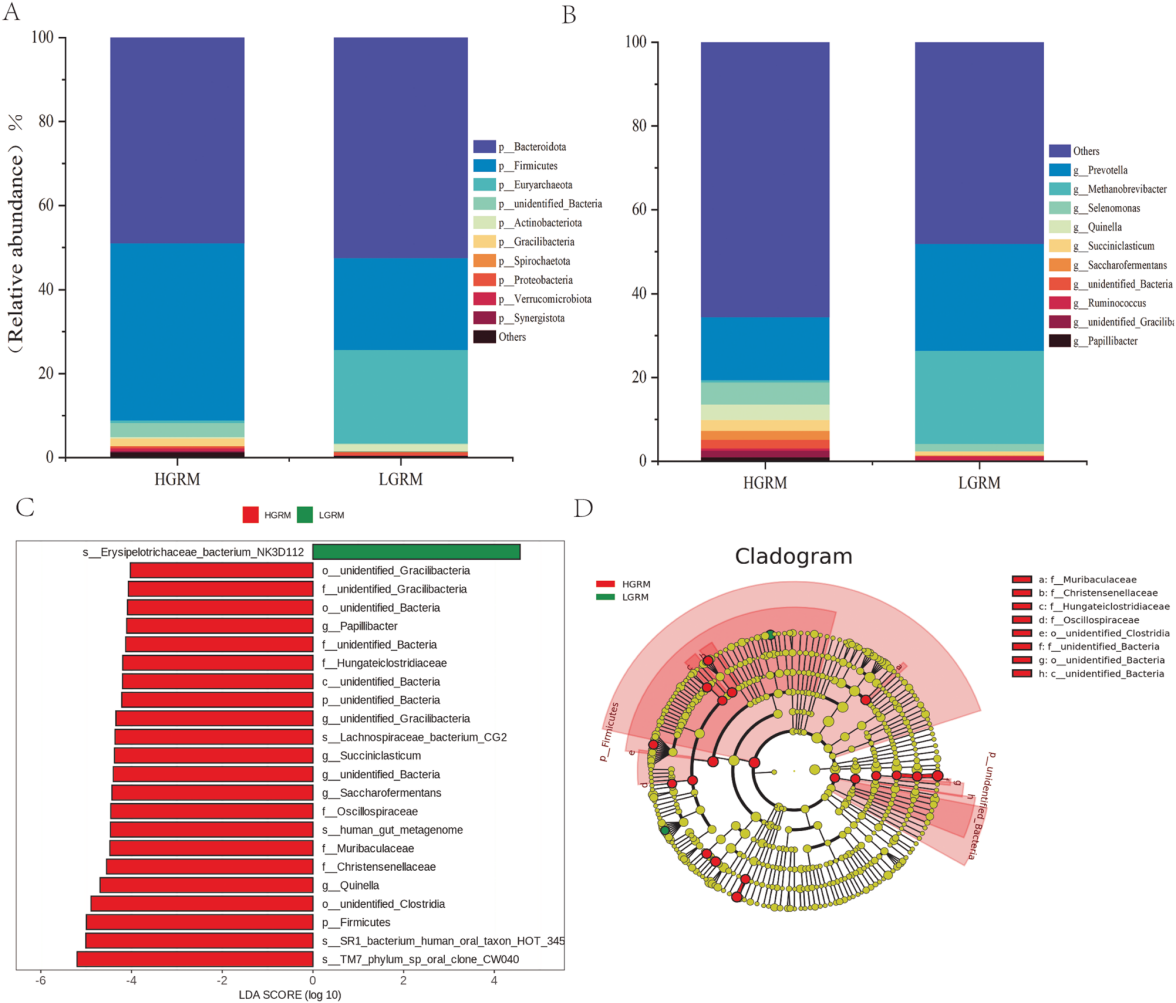
Comparison of changes in rumen microbiota composition between Xizang and low-altitude goats. (**A**) Composition of microbiota phylum level in Xizang and low-altitude goats. (**B**) Composition of microbiota genus level in Xizang and low-altitude goats. (**C**) Cladogram generated from the LEfSe analysis indicating the phylogenetic distribution from phylum to genus levels of the microbiota of Xizang goats and low-altitude goats. (**D**) Histogram of LDA scores to identify differentially abundant bacterial genera between Xizang goats and low-altitude goats

At the microbial phylum classification level, the relative abundance of TOP10 rumen microbiota of Xizang goats and low-altitude goats was demonstrated by Taxonomy analysis as shown in Fig. 3A. Among these, *Bacteroidota* accounted for about 49.0% of the Xizang goats and was the dominant phylum, followed by *Firmicutes*, *Gracilibacteria*, and *unidentified_Bacteria*, which accounted for about 42.1%, 1.8%, and 3.3%, respectively. In low-altitude goats, the relative abundance share of *Bacteroidota* increased by about 3.5%, while *Euryarchaeota* increased by approximately 21.7%. In contrast, the relative abundance share of *Firmicutes* decreased by about 20.3% compared to Xizang goats. At the microbial genus classification level, as depicted in Fig. 3B, the common genera in Xizang goats included *Prevotella* (15.0%), *Succiniclasticum* (2.6%), *Quinella* (3.7%), and *Selenomonas* (5.2%). In contrast, the common genera in low-altitude goats were *Prevotella* (25.4%) and *Methanobrevibacter* (22.2%).

LEfSe (LDA Effect Size) is an analytical tool for discovering and interpreting high-dimensional biomarkers that can be used to make comparisons between two or more subgroups. This study employed LEfSe to assess whether specific bacterial taxa were differentially enriched in Xizang goats compared to low-altitude goats. Using a log LDA score cutoff of 4, the study identified 23 differentiating genera as key differentiators (Fig. 3C). Twenty-two genera, including *Firmicutes*, *Christensenellaceae*, *Succiniclasticum*, *Saccharofermentans*, *Oscillospiraceae*, *Muribaculaceae*, *Papillibacter*, and *Lachnospiraceae_bacterium_CG2*, among others were significantly enriched in Xizang goats. In contrast, only *Erysipelotrichaceae_bacterium_NK3D112* was significantly enriched in low-altitude goats. Branch diagrams representing the taxonomic hierarchy of rumen microbiota from phylum to species level showed significant differences in phylogenetic distributions between the microbiota of Xizang goats and low-altitude goats (Fig. 3D). The results indicated that there were significant differences in rumen microbiota composition between low-altitude goats and Xizang goats.The significant enrichment of the microbiota may be beneficial for the adaptation of Xizang goats to the plateau environment.

### Correlation analysis between Rumen Microbiota and climate

**Fig. 4:**
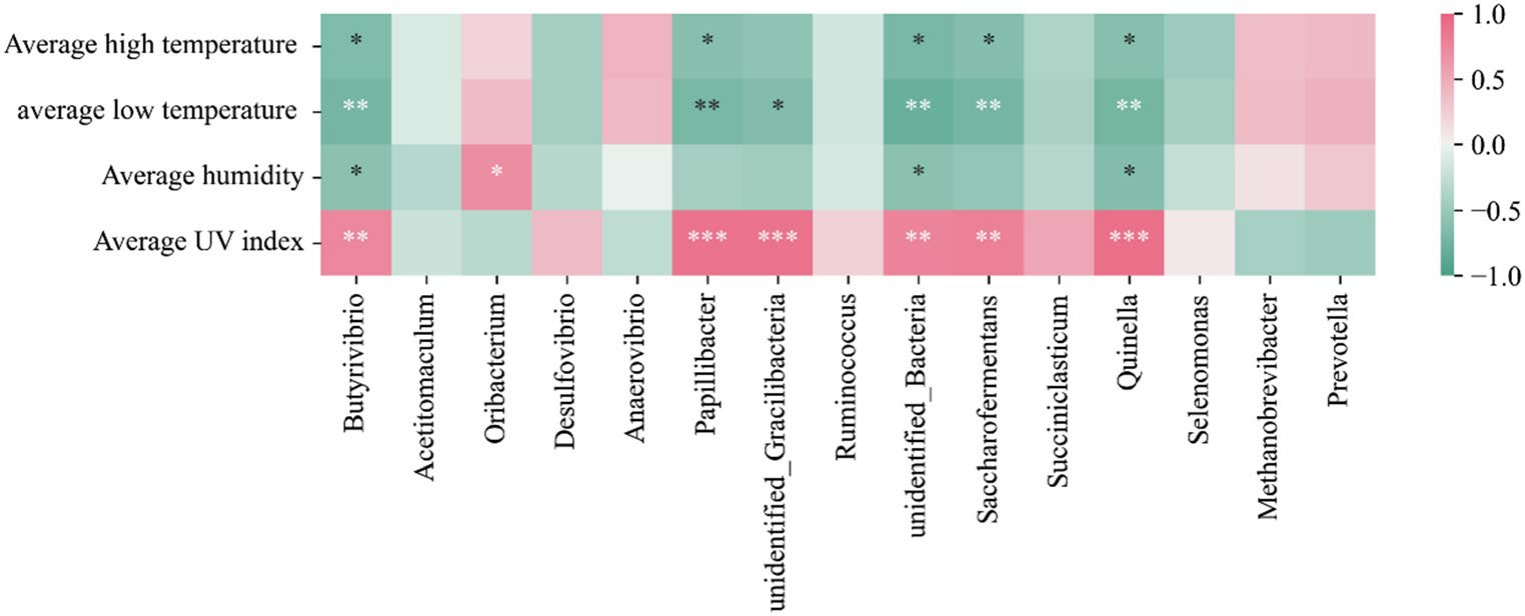
Correlation analysis between rumen microbiota and climate. ⁎ denotes significant, ⁎⁎ denotes highly significant, ⁎⁎⁎ denotes extremely significant

The current study used host-climate correlations and heat maps using Spearman correlation analysis to highlight the relationship between rumen microbiota and climate as well as their interactions in the adaptation of Xizang goats to the plateau environment. We choose to connect the relative abundance data of the microbiota with the climate data from April to September, given that our sample was taken in mid-June. As shown in Fig. 4, we observed significant positive correlations (*p* < 0.001) between the average UV index of the climate and *Papillibacter*, *Quinella*, and *unidentified Gracilibacteria*. Additionally, the average humidity showed significant positive correlations (*p* < 0.05) with *Oribacterium*, *Quinella*, and *Butyrivibrio*. Furthermore, the average low temperature exhibited a significant negative correlation (*p* < 0.01) with *Saccharofermentans* and *Papillibacter*, among others, while the average high temperature displayed a significant negative correlation (*p* < 0.05) with *unidentified Bacteria*, *Saccharofermentans*, and *Quinella*, among others. Interestingly, we discovered that numerous bacterial families—*Papillibacte*, *Quinella*, and *Saccharofermentans*, for example—that exhibited substantial correlations with climatic parameters also had significant variations in relative abundance between Xizang goats and low-altitude goats. As a result, Xizang goats had considerably higher concentrations of *Papillibacter*, *Quinella*, and *Saccharofermentans*, which may help Xizang goats adapt to the extreme cold, low temperature, and strong radiation seen on the plateau.

### Analysis of DEGs

**Fig. 5:**
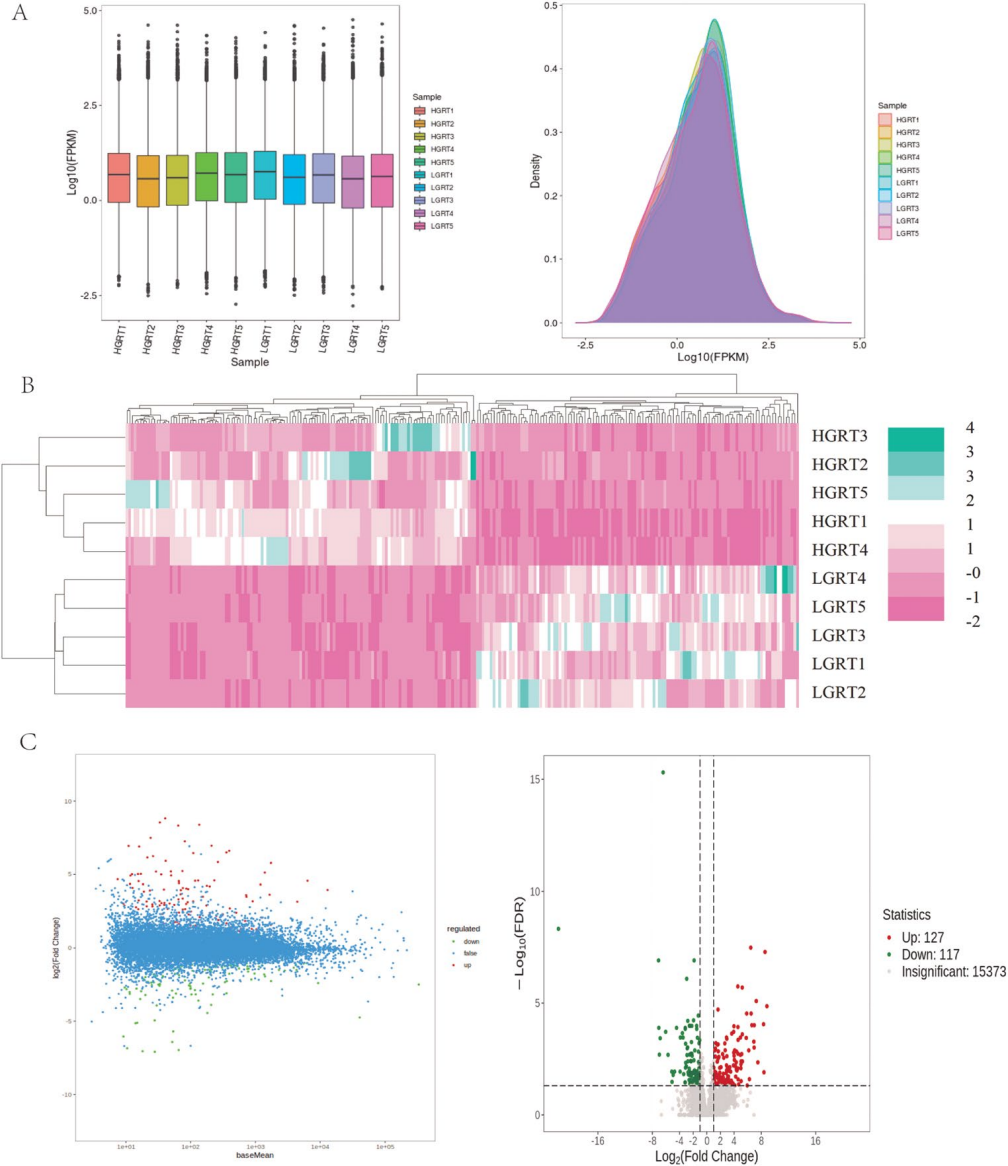
DEGs in Xizang and low-altitude goats. (**A**) Trends in overall gene expression levels and expression in Xizang and low-altitude goats. (**B**) Clustering of DEGs in Xizang goats and low-altitude goats. (**C**) Volcano maps of DEGs in Xizang and low-altitude goats. Xizang goats represent HGRT, Low-altitude goats represent LGRT

In Fig. 5A, boxplots and dendrograms illustrate the overall expression level of the sample genes as well as the trend of gene abundance with the change in expression. Transcriptome sequencing of the rumen epithelial tissues of Xizang goats and low-altitude was conducted for this study. The results showed that 244 genes were differentially expressed in the rumen epithelial tissues of the two groups, with 127 genes up-regulated and 117 genes down-regulated (Fig. 5C). Further cluster analysis (Fig. 5B) revealed that genes with high and low expression levels were clustered together in the rumen tissue samples of Xizang goats and low- altitude goats, respectively. We screened 31 DEGs using the KEGG pathways (HIF-1 signalling system, metabolic pathways, glutathione metabolism, etc.) and plateau adaptation literature as a basis. Table 2 lists 31 DEGs in the rumen tissues of Xizang goats versus low-altitude goats, which may be involved in regulating plateauing (17 up-regulated and 14 down-regulated in the expression).

**Table 2:** DEGs in the rumen tissue of Xizang and low-altitude goats that may be associate with plateau adaptation

Gene symbol	log_2_FC	Gene symbol	log_2_FC	Gene symbol	log_2_FC
*FMO4*	1.666160657152	*KLK11*	5.028603511316	*IRAK2*	1.658030138
*DPEP3*	-2.9558349230	*PFKFB3*	1.53365921717	*PRKCE*	-1.247235543
*LFNG*	-1.4795011690	*COQ7*	-1.2155281382	*PSMC3IP*	1.272835922
*SLC6A4*	-3.1699806090	*NOS2*	4.32482251878	*GPX3*	2.701480361
*ABCA12*	5.2085317189	*TKT*	-1.11446292603	*AMACR*	-1.765407414
*MYH2*	4.9346071275	*EPHB6*	3.17362673701	*SLC26A9*	5.2310992941
*PTGES*	2.8926980708	*SLC27A2*	-2.75378282529	*S100A8*	3.158960510
*CHST2*	-1.3726226148	*FPGS*	-1.80690217049	*STEAP4*	2.4229746835
*KLK12*	5.7875456760	*IDNK*	-1.04126356779	*NEDD9*	-1.429587947
*DNAJC9*	-1.1417343037	*ABCA10*	2.418474166101	*COX1*	-2.493679237
*PCSK7*	1.2761049956				

### Functional enrichment analysis of DEGs

**Fig. 6:**
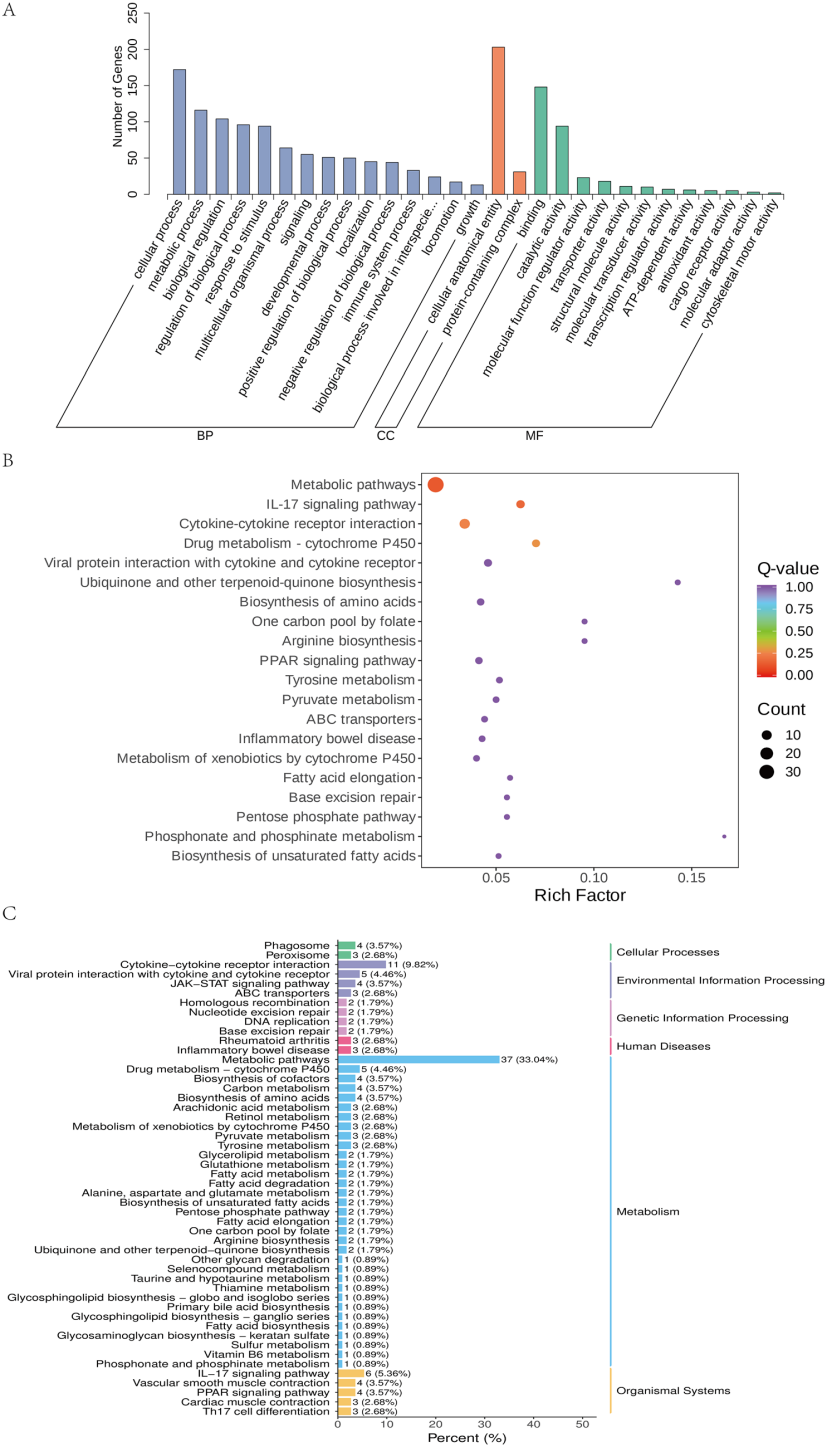
Functional enrichment analysis of DEGs. (**A**) Gene ontology (GO) classification map. (**B**, **C**) KEGG enrichment map of all differential genes

Differently expressed genes were annotated in Gene Ontology (GO) and Kyoto Encyclopedia of Genes and Genomes (KEGG) databases. In the GO database, the annotated genes were categorized into three groups: biological process (BP), cellular component (CC), and molecular function (MF). As shown in Fig. 6A, GO functional categorization revealed that most of the DEGs associated with BP were mainly enriched in metabolic process (GO:0008152), cellular process (GO:0009987), and biological regulation (GO:0065007), regulation of biological process (GO:0050789) and response to stimulus (GO:0050896). The DEGs associated with CC were mainly enriched in cellular anatomical entity (GO:0110165), protein-containing complex (GO:0032991), multicellular organismal process(GO:0032501)and positive regulation of biological process (GO:0048518). Fewer DEGs were annotated in MF category, primarily associated with binding (GO:0005488), catalytic activity (GO:0003824), molecular function regulator activity (GO:0098772), and molecular transducer activity (GO:0060089). The statistics of GO enrichment results showed that BP were enriched with the highest number of differential genes, followed by MF and CC.

DEGs were analyzed for KEGG pathway enrichment to determine if significant differences occurred in a given pathway. As shown in Fig. 6(B-C), the most significantly enriched pathways included Metabolic pathways, such as Drug metabolism - cytochrome P450 (4.46%), Carbon metabolism (3.57%), Arachidonic acid metabolism (2.68%), Retinol metabolism (2.68%) and Fatty acid metabolism (1.79%), among others. The next pathways that were significantly enriched included Cytokine-cytokine receptor interaction (9.82%) and IL-17 signaling pathway (5.36%).

### RT-qPCR validation analysis

**Fig. 7:**
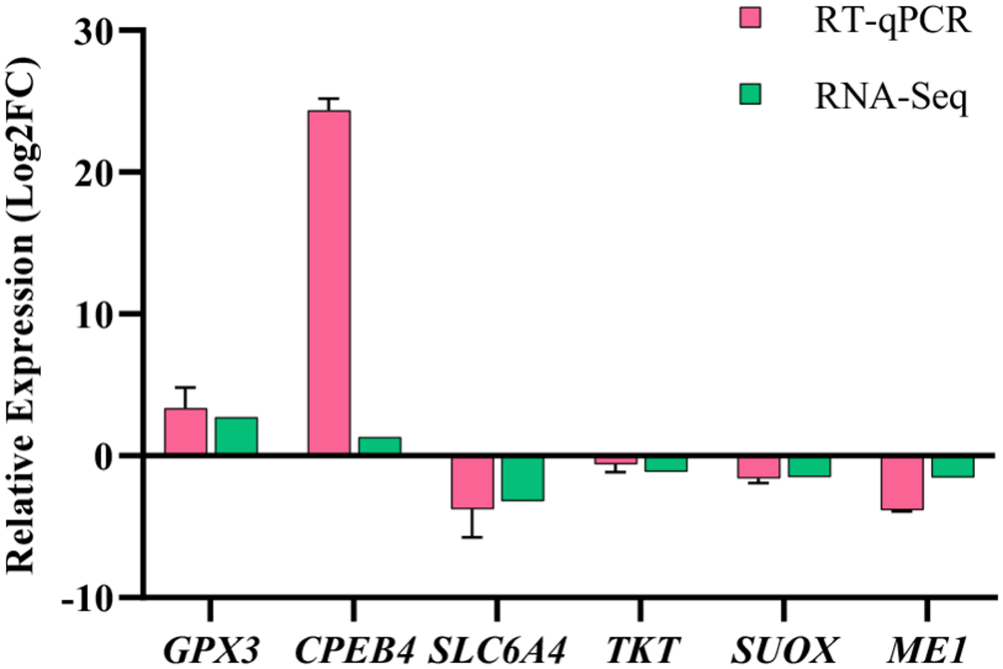
Verification of DEGs using qRT-PCR

To validate the reproducibility and repeatability of gene expression levels in RNA-seq, six DEGs (*TKT*, *SLC6A4*, *CPEB4*, *GPX3*, *SUOX*, *ME1*) were randomly chosen and validated via qRT-PCR. The results as depicted in Fig. 7 were consistent with the RNA-seq results.

### Interactions between Rumen plateau adaptation-related host genes and rumen microbes

**Fig. 8:**
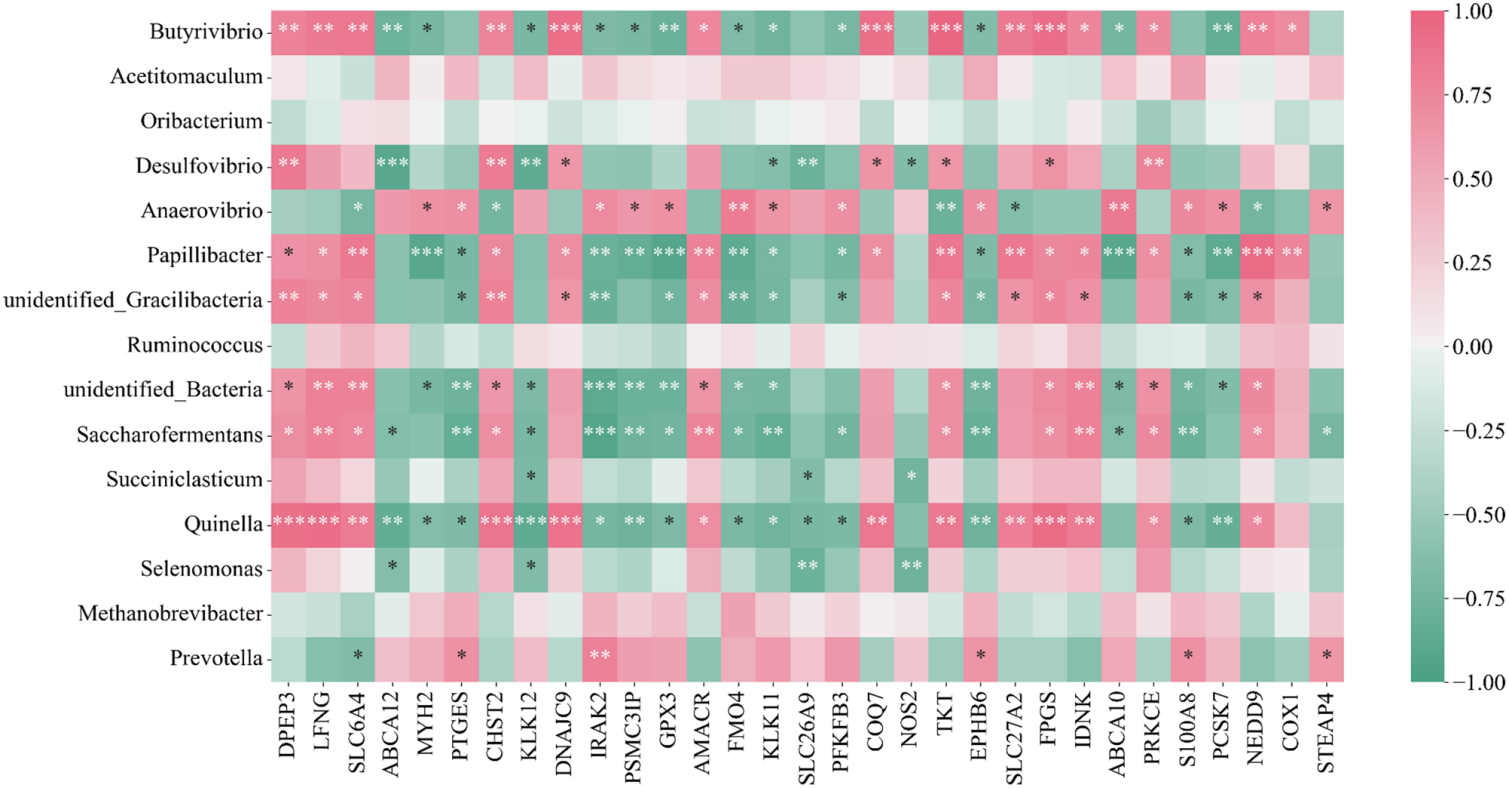
Interactions between rumen plateau adaptation-related host genes and rumen microbes. ⁎ denotes significant, ⁎⁎ denotes highly significant, ⁎⁎⁎ denotes extremely significant

To investigate the relationship between host genes and microorganisms in the rumen and their potential role in the mechanism of plateau adaptation in Xizang goats, we analyzed the correlation between host genes and microorganisms. We selected 31 relevant genes from 244 DEGs that might be involved in plateau adaptation for Spearman’s correlation analysis with the top 15 microbiota at the genus level. Interestingly, most of the taxa significantly associated with genes also exhibited significant differences in abundance between Xizang goats and low-altitude goats. We visualized all the correlations between taxon abundance and host gene expression in Fig. 8. We also identified some genes that showed positive correlation (*P* < 0.001) with rumen microbiota, including three genes-*GPX3*, *ABCA10*, and *KLK11*-showed positive correlations with *Saccharofermentans*, *PCSK7* and *Butyrivibrio*, and *COX1* and *Prevotella*. Several significant negatively correlated genes and microbiota were also identified, such as *COQ7* and *Quinella* and *TKT* with *unidentified_Gracilibacteria* as shown in Fig. 8.

## Discussion

Known as the Roof of the World, the Xizang Plateau is the highest plateau on Earth [19]. The climate of Xizang Plateau, considered sensitive and vulnerable to global climate change, varies significantly across regions.It is characterized by low temperatures, lengthy winters, plenty of sunshine, and a broad variety of daytime and nighttime hours [20]. The Xizang Plateau is the world’s alpine and high-altitude region with the greatest biodiversity, as well as a significant center of origin and differentiation of the world’s mountain biological species [21, 22]. One of the distinctive species of the Xizang Plateau, the Xizang goat, has persistently thrived and reproduced in its population despite the challenging environment of the plateau. This may be directly linked to the interactions between host genomes and the features of tissues and organs, as well as the diversity and composition of rumen microbes [18, 23]. One of the key elements influencing an animal’s ability to grow, develop, and adapt to its surroundings is the climate [24]. In the current study, it was observed that the average UV index was much lower at low altitudes than at high altitudes, while the average high and low temperatures as well as humidity were significantly greater at low altitudes than at high altitudes.This suggests that Xizang goats live in exceptionally hostile settings. The current study extensively used analytical techniques like rumen transcriptomics and microbiology to explore the interactions between rumen hosts and microbiota as well as the relationship between climate and rumen microbiota in Xizang goats in order to further explore the regulatory mechanisms of environmental adaptation in these goats at high altitudes.

The rumen is the largest digestive organ found in ruminants and serves as a vital home for bacteria and other microbes [7]. The Chao1 and Shannon indices, which gauge the microbiota’s richness and variety, respectively, are the two main components of the comprehensive index used to assess the evenness and richness of microbiota, known as ruminal microbiota diversity. The richness and diversity of the microbiota in the sample increases with increasing Shannon and Chao1 indices. The present investigation reveals that the rumen microbiota of Xizang goats had significantly greater values of the Chao1 index, Shannon index, PD_whole_tree, and ACE index in comparison to goats from low altitudes. The current study results are consistent with the adaptation of yak rumen microorganisms at high and low altitudes.It has been demonstrated that the indices of observed species,such as Chao1, ACE, and Shannon, were significantly higher in yak rumen microbiota at high altitude than at low altitudes [25]. The PCoA analysis revealed a significant difference in β-diversity between low-altitude goats’ rumen microbiota and that of Xizang goats. In studies of rumen bacterial communities, the rumen microbial diversity of yaks was found to increase with altitude at three different levels: 2800 m (low altitude), 3700 m (middle altitude), and 4700 m (high altitude) on the Xizang Plateau [26]. In contrast, the current study found that the diversity of rumen microbiota of Xizang goats was significantly higher than that of goats at low altitudes. A strong metabolism and stability have been found to be closely correlated with large microbial diversity [27]. Consequently, we postulated that the rumen community of Xizang goats residing at high altitudes would possess enhanced metabolic stability and capability, enabling them to meet the energy requirements of their frigid and alpine environments. A comparative analysis of the relative abundance of rumen microbiota composition in dairy cows, cattle, and yaks revealed significant variations [28], whereas the current study showed differences between Xizang goats and low-altitude goats in terms of the relative abundance of rumen microbiota composition. The microbial communities were primarily dominated by *Bacteroidote* and *Firmicutes*, according to Kumar et al.‘s investigation of the whole genome of the cecum microbiome of native Ethiopian chickens from two geographically distinct regions: the Amhara zone (Menz Gera Midir, 3300 m) and the Afar zone (Dulecha, 730 m above mean sea level) [29]. At the phylum level, this study discovered that *Bacteroidote* and *Firmicutes* were the predominant microbiota in the rumen of Xizang and low-altitude goats, respectively, whereas *Euryarchaeota*, *Firmicutes*, and *Bacteroidote* were the predominant microbiota in the rumen of low altitude goats.The relative abundance of ruminal *Firmicutes* in Xizang goats increased by 20.3% as compared to goats from low altitudes. *Firmicutes* to *Bacteroidote* ratios have been employed as a crucial metric to assess how microorganisms affect the host’s energy needs. *Firmicutes* play a major role in the breakdown of fibrous materials, while *Bacteroidotes* primarily break down non-fibrous materials [30, 31]. This process is critical to the nutritional metabolism of ruminants. Considering this, the current study postulated that the larger relative abundance of *Firmicutes* in the rumen of high-altitude Xizang goats is involved in the breakdown of cellulosic materials, providing the host with more energy to meet the demands of adapting to the plateau habitat. *Prevotella* is the predominant genus in the rumen of Xizang goats, with *Quinella*, *Selenomonas*, and *Succiniclasticum* being other genera.*Prevotella* is a bacterium that breaks down proteins in the rumen and gastrointestinal tract of ruminants. It primarily breaks down the hemifibrous parts of the ration, and the genus produces a wide range of intricate enzymes that assist in the breakdown of pectin and non-fiber polysaccharides [32]. Research on the microbial community structure and population dynamics of the rumen epithelium during the development of young ruminants suggests that, *Succiniclacticum* may play a role in facilitating the maturation of the rumen epithelium [33].As a characteristic rumen bacterium, *Quinella* reduces CH4 emissions as its relative abundance rises [34]. *Succiniclasticum* and *Quinella* were found to be significantly more abundant in the rumen of Xizang goats than in goats belonging to low altitudes. This suggests that they support the development of the rumen in Xizang goats and reduce the production of the harmful gas CH4, further enhancing the goats’ ability to adapt to the plateau. The current study considered host-climate connections and analyzed them using the Spearman correlation method to further elucidate the relationship between rumen microbiota, as well as climate and their interactions in the adaptation of Xizang goats to the plateau environment. In the current study,it was discovered that there were significant negative correlations between average low temperature and *Saccharofermentans* and *Papillibacter* et al., the average high temperature was significantly negatively correlated with *Saccharofermentans* and *Quinella*. There were highly significant positive correlations between the average UV index of climate and *Papillibacter* and *Quinella*; significant positive correlations between average humidity and *Oribacterium*, but significant negative correlations with *Quinella*. Itwas interesting to note that we discovered significant differences in the relative abundance of some bacterial families (including *Papillibacter*, *Quinella*, and *Saccharofermentans*) between low-altitude goats and Xizang goats, which are significantly correlated with climate conditions.Consequently, the current study conjectured that *Papillibacter*, *Quinella*, and *Saccharofermentans* were markedly enriched in the rumen of Xizang goats, potentially advantageous for the goats’ adaptation to the severe climate of high altitude, low temperature, and high radiation.

Furthermore, genes like *THEK*, *VTG 1*, *SGK*, and *CDK 2* were found to be highly involved in host adaptation to different climatic conditions in one study that analysed the liver transcriptome of Korean commercial chickens in two different environments using RNA-seq to investigate their role during adaptation to different climatic conditions [35]. Therefore, interactions between the host transcriptome and climate are crucial for environmental adaptability. In a comparative analysis of the transcriptomics and proteomics of heart tissues from Tibetan and Yorkshire pigs raised on plateaus and lowlands, Zhang et al. identified genes enriched in the VEGF signalling pathway (*ERK 2*, *A2 M*, *FGF 1*, *CTGF*, and *DPP 4*), the HIF-1 signalling pathway (*NPPA*, *ERK 2*, *ENO 3*, and *EGLN 3*), and hypoxia-associated processes (*ESTAB*, *EGLN 3*, *TGFB 2*, *DPP 4*, and *ACE*) as significant candidates for highland adaptation in Tibetan pigs raised on plateaus and lowlands [36]. Gou et al. conducted a comparison of dog breeds at different altitudes and discovered major signals of population differentiation at hypoxia-associated gene loci, such as *β*-hemoglobin clusters and endothelium Per-Arnt-Sim (PAS) structural domain protein 1 (EPAS 1) [37]. Rumen epithelial tissues of high-altitude Xizang goats and low-altitude goats were subjected to transcriptome sequencing analysis in this study. The results showed that 244 genes were differentially expressed in the rumen epithelial tissues of the two groups of goats, with 127 genes showing up-regulation and 117 genes showing down-regulation. Long-term high altitude life, confronted with low oxygen, low pressure, low temperature, and intense UV light, can cause oxidative stress in animals to varying degrees, leading to increased nitrogen and reactive oxygen production [38, 39]. Xizang goats need to have physiological and genetic responses to oxidative stress since they endure an extensive period of time in low-oxygen conditions. In line with the lower *GPX* activity in Tibetans living on the plateau, it was found that *GPX 1* was expressed at significantly lower levels in Tibetan sheep when compared to lake sheep [40]. This suggests that lake sheep, which are more susceptible to oxidative stress than Tibetan sheep, have higher antioxidant ability [41]. In addition to being critical for preserving the redox system’s homeostasis, glutathione peroxidase 3 (*GPX3*) is also involved in anti-oxidative stress, inflammatory signaling, and metabolic disorders [42]. The only extracellular *GPX* in the oxidoreductase family that catalyzes the reduced glutathione-mediated detoxification of hydroperoxides and soluble lipid hydroperoxides is glutathione peroxidase 3 (*GPX3*), a selenoprotein [43]. Since Xizang goats expressed more *GPX3* than did goats at lower altitudes, we reasoned that *GPX3* might be crucial to highland Xizang goats’ ability to withstand UV radiation. *PFKFB3*, also known as 6-phosphofructo-2-kinase/fructose-2,6-bisphosphatase 3, is an essential enzyme that facilitates the breakdown of glucose [44]. The gastrointestinal tract expresses *SLC26A9*, one of the 11 members in the *SLC26A* family of anion transport proteins, at a high level [45]. Previous research has demonstrated that *SLC26A9* mediates bicarbonate secretion, is expressed in mouse gastric surface epithelial cells, and plays a protective role in shielding the gastric mucosa [46, 47]. The ion transport pathway is primarily mediated by the *SLC26A9* gene, whose expression level is positively associated with the width and length of the papilla [48]. The hypothesis was that *SLC26A9* protected the rumen of Xizang goats from the stimulation of the harsh plateau environment and further enhanced their adaptation to the environment. The results demonstrated that the expression level of *SLC26A9* in the rumen of Xizang goats was higher than that of low-altitude goats. *ARRDC4* is a crucial regulator of glucagon signaling and glucose homeostasis, as evidenced by the reduced glucose levels and decreased glucagon response observed in *ARRDC4* knockout mice [49]. The results of the current study showed that the expression of *ARRDC4* was higher in the rumen of Xizang goats than in goats raised at low altitudes. This suggests that *ARRDC4* regulates glucose homeostasis in the rumen of Xizang goats during digestion and absorption. The findings of the GO enrichment analysis revealed that the cellular components were enriched in both biological processes and molecular functions among the DEGs. Numerous categories including cell shape, binding, and bioregulation, indicate that Xizang and low-altitude goats differ in these areas. Utilizing KEGG pathway analysis to better understand the biological roles of these genes by examining the metabolic pathways, we can obtain information on some of the signaling pathways implicated in DEGs [50]. Transcriptome analysis uncovered the molecular regulatory mechanisms of plateau oxygen tolerance in Xizang goats. It was discovered that the key pathways for Xizang sheep to adapt to plateau, hypoxia, and UV light resistance may include the relaxation signaling pathway, protein digestion and absorption, platelet activation, thyroid hormone synthesis, actin cytoskeleton regulation, arachidonic acid metabolism, glutathione metabolism, and nucleotide excision repair [51]. The current study discovered that the metabolism of fatty acids, arachidonic acid, and the interaction between cytokines and cytokine receptors may be important routes in the Xizang goats’ adaptation to the environment of the plateau. At the cellular and organ levels, fatty acid metabolism—which involves the production, absorption, oxidation, and derivatization of adipose fatty acids from the head—is crucial [52]. One of the most prevalent and abundant ω-6 polyunsaturated fatty acids is arachidonic acid, which is found in esterified form in the membrane phospholipids of all mammalian cells. Several phospholipases release arachidonic acid from phospholipids in response to different stimuli that either activate or inhibit them [53]. Arachidonic acid metabolism is a finely tuned system for vascular health and disease due to the abundance of Arachidonic acid converting enzymes, downstream synthases and isoenzymes, transmembrane receptors, and the specificity of their tissue expression [54]. The adaptation of Xizang goats to hypoxia, altitude, and UV resistance may be attributed to these routes.

We found multiple associations between differently expressed rumen epithelial genes and rumen bacteria in Xizang and low-altitude goats by integrating the rumen microbiome and host gene expression patterns. Evidence from the relationship between host gene expression and the composition of the gut microbiome in non-mammalian vertebrate species, suggests that gene expression may act as a mediator in these relationships between microbial communities and host function [55]. In this study, the only extracellular *GPX* in the oxidoreductase family that catalyzes the detoxification of hydroperoxides and soluble lipid hydroperoxides via reductive glutathione, *Anaerovibrio*, was found to have a positive and significant correlation with *GPX3* [43]. Conversely, a reduction in *Anaerovibrio* may result in a reduction in lipolysis [56], suggesting that *GPX3* genes and *Anaerovibrio* may cooperate to support the development of rumen epithelium to improve the high plateau acclimatization of Xizang goats. Research on the impact of dietary nitrate addition on rumen fermentation and microbial communities in goats suggests that *Selenomonas* and *SLC26A9*. *Selenomonas* may play a crucial role in the reduction of nitrate and nitrite in the rumen [57]. The stomach mucosa is shielded, and bicarbonate secretion is mediated by *SLC26A9*, which is expressed in mouse gastric surface epithelial cells [47, 49]. Thus, a shared function of the *Selenomonas* and *SLC26A9* genes in safeguarding the rumen of Xizang goats is theorized. *Butyrivibrio*, expressed in the majority of tissues, including blood vessels, the stomach, and platelets, and involved in platelet aggregation, vasodilation and contraction, as well as the regulation of gastric mucosal blood flow to maintain the stability of physiological functions of cells, tissues, and organs [58, 59], was found to have a significant positive correlation with the highly expressed *COX1* gene [60]. Additionally, the rumen *Butyrivibrio* is an important degrader and user of lignocellulosic plant materials. These results suggest that the ability of Xizang goats to adapt to the plateau may be enhanced by genes associated with plateaus and the accompanying rumen microorganisms.

## Conclusions

In summary, our study reveals notable modifications in the control of the rumen transcriptome and rumen microbiota in plateau-acclimated Xizang goats at high altitudes. We found that both genes that enhance plateau resistance and those associated with antioxidant activity were upregulated. Furthermore,alterations in the *Papillibacter*, *Quinella*, and *Saccharofermentans* microbiomes were noted, potentially aiding Xizang goats in adapting to the harsh climate conditions on the plateau. In conclusion, our research provides insights into potential interactions between host genes and rumen microbiota in Xizang goat’ rumen, offering potentia biomarkers for research on plateau adaptability.

### Electronic supplementary material

Below is the link to the electronic supplementary material.

Supplementary Material 1

## Data Availability

All data in this study are available upon request by contact with the corresponding author. Sequencing data for Xizang and low altitude goats have been submitted to the NCBI Sequencing Read Archive (SRA) under Biological Programs PRJNA1036847 and PRJNA1041396.
