# Supplementary material for: Coping with extremes: the rumen transcriptome and microbiome co-regulate plateau adaptability of Xizang goat
Source: BMC Genomics. 2024 Mar 7;25:258. doi: 10.1186/s12864-024-10175-8 (PMC10921577; doi:10.1186/s12864-024-10175-8)
Supplement: Supplementary file 1 — Supplementary Material 1 [file 12864_2024_10175_MOESM1_ESM.pdf]

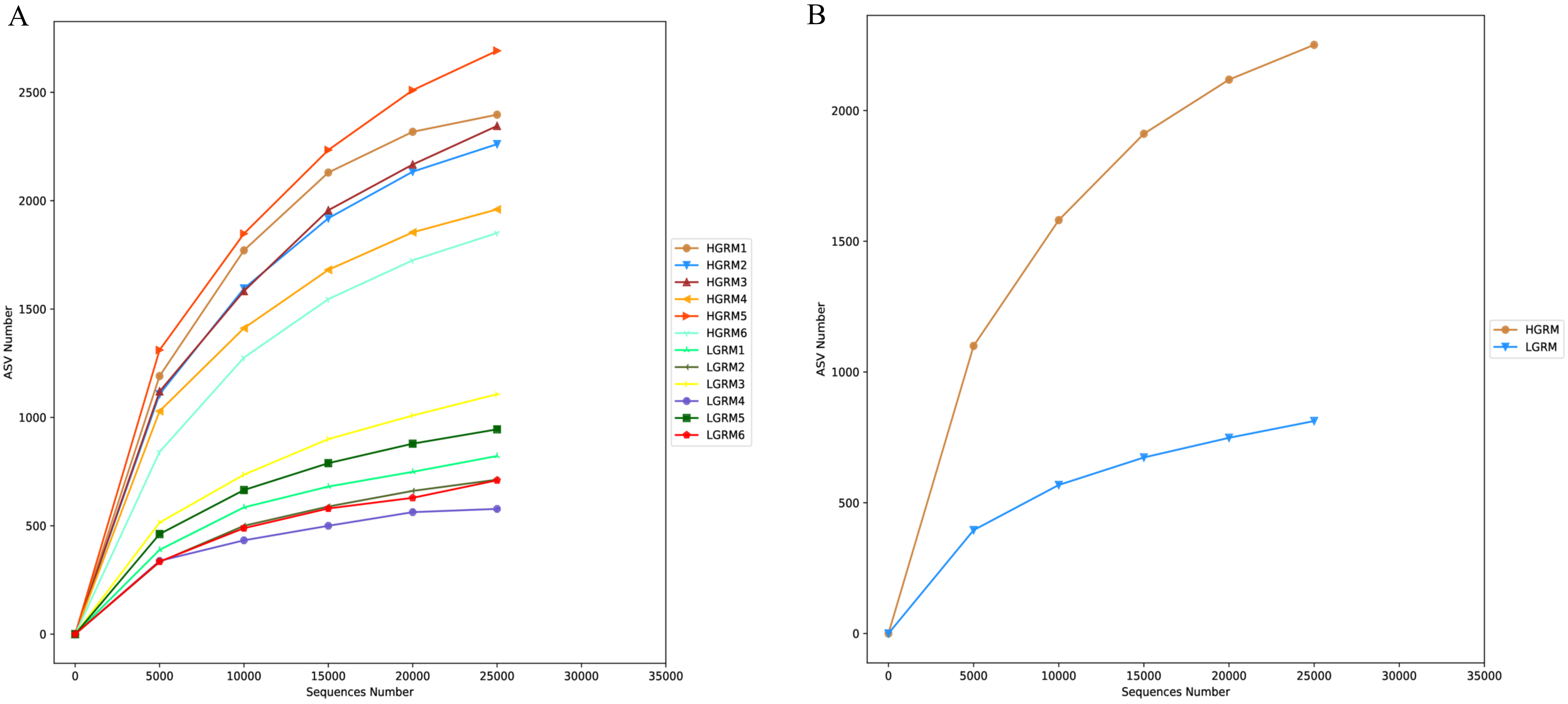

Fig.s 1 Rarefaction curve. (A) Rarefaction curve for each sample based on ASV. (B) Rarefaction curve for each group based on ASV
